# Coronary Artery Anomalies Revisited: Description of the Types, Pathophysiology and Treatment Options Based on Latest Guidelines

**DOI:** 10.3390/jcm15134959

**Published:** 2026-06-25

**Authors:** Alexandre Genoud, Ines Portugal, Nicolas Murith, Jean-Francois Deux, Tornike Sologashvili, Christoph Huber, Mustafa Cikirikcioglu

**Affiliations:** 1 Faculty of Medicine, University of Geneva, 1205 Geneva, Switzerland; alexandre.genoud@etu.unige.ch (A.G.);; 2 Cardiovascular Surgery Division, Department of Surgery, University Hospitals of Geneva, 1205 Geneva, Switzerland; 3 Radiology Division, Department of Diagnostic, University Hospitals of Geneva, 1205 Geneva, Switzerland

**Keywords:** coronary artery anomalies, anomalous aortic origin of a coronary artery, AAOCA, anomalous left coronary artery from the pulmonary artery, ALCAPA, sudden cardiac death, myocardial ischemia, risk stratification, coronary computed tomography angiography, surgical management

## Abstract

Coronary artery anomalies (CAAs) are a rare but clinically significant group of congenital abnormalities that are associated with myocardial ischaemia, malignant arrhythmias and sudden cardiac death, particularly in young people and athletes. Despite increasing recognition of these conditions and advances in cardiovascular imaging, there are still significant challenges regarding their classification, risk stratification and management, particularly with respect to surgical indications. This review provides a comprehensive overview of the current evidence on the classification, pathophysiology, diagnosis and management of CAAs, with a particular focus on surgical decision-making and contemporary guideline recommendations. A systematic literature search was conducted up to February 2025 using PubMed and Google Scholar. Priority was given to international guidelines, consensus statements, systematic reviews, meta-analyses and large observational studies. CAAs encompass a broad spectrum of anatomical variants and clinical presentations. Among these, anomalies of coronary origin are the most extensively studied. Surgical management is well established for coronary arteries arising from the pulmonary artery, particularly for anomalous left coronary artery from the pulmonary artery (ALCAPA). Substantial advances have also been made in the diagnosis, risk stratification, and treatment of anomalous aortic origin of a coronary artery (AAOCA), which has become a major focus of contemporary guideline recommendations. For patients with AAOCA, surgical correction, including unroofing, coronary reimplantation or coronary artery bypass grafting, is recommended for individuals with symptoms and/or high-risk anatomical features. In contrast, the diagnosis and management of myocardial bridging, coronary artery fistulas, and coronary artery ectasia remain controversial, with considerable variability in the indications for medical, percutaneous, and surgical treatment. The management of CAAs is an evolving field. While there is consensus for a limited number of anomalies, most cases still require individualised decision-making. It is essential to develop standardised diagnostic frameworks, improved risk stratification tools and outcome-based management criteria. A multidisciplinary, evidence-based approach involving cardiologists, cardiac imagers, interventional cardiologists and cardiac surgeons is crucial in order to optimise patient outcomes and reduce the risk of adverse cardiovascular events, including sudden cardiac death.

## 1. Introduction

Coronary artery anomalies (CAAs) are a diverse group of congenital variations in the anatomy of the coronary arteries, involving abnormalities of their origin, course or termination. They are estimated to affect less than 1% of the general population. The clinical presentation can range from individuals with incidental diagnoses who are asymptomatic, to patients presenting with myocardial ischaemia, arrhythmias, myocardial infarction or even sudden cardiac death (SCD). CAAs are recognised as an important cause of SCD in young people and are one of the leading cardiovascular causes of death among competitive athletes [1,2].

Despite substantial advances in understanding CAA anatomy and associated clinical risks, important gaps remain in our knowledge of their natural history, long-term prognosis and optimal management. Furthermore, the genetic and developmental mechanisms underlying these anomalies are not fully understood.

This review aims to summarise the available literature on the main CAA subtypes, providing a structured overview of their classification, pathophysiology, clinical presentation, diagnostic evaluation and management. Particular emphasis is placed on contemporary guideline recommendations and surgical decision-making.

A literature search was conducted up to February 2025 using PubMed and Google Scholar. Relevant Medical Subject Headings (MeSH) and free-text keywords related to coronary artery anomalies, clinical presentation, diagnostic imaging, risk stratification and management were employed. Boolean operators were used to optimise search sensitivity. Priority was given to contemporary clinical practice guidelines, consensus statements, systematic reviews, meta-analyses and major observational studies. Articles for which full-text access was unavailable were excluded.

## 2. Normal Coronary Artery Anatomy

In order to better contextualise coronary artery anomalies, it is first necessary to define normal coronary anatomy. According to the classification proposed by Angelini et al., coronary anatomical patterns that occur in more than 1% of the general population are considered normal variants, while those that occur less frequently are regarded as coronary artery anomalies [1]. The qualitative and quantitative criteria used to characterise normal coronary anatomy are summarised in Table 1 [1].

The epicardial coronary circulation comprises three main vessels: the right coronary artery (RCA), which originates from the right sinus of Valsalva; and the left anterior descending (LAD) and left circumflex (LCX) arteries, which arise from the left main coronary artery (LMCA), which in turn originates from the left sinus of Valsalva.

The RCA runs through the right atrioventricular groove, supplying the right atrium, right ventricle and portions of the interventricular septum. Depending on coronary dominance, it also supplies part of the inferior wall of the left ventricle. Major branches include the conus artery, the sinoatrial nodal artery, the right marginal artery, the atrioventricular nodal artery, the posterior descending artery (PDA) and the posterolateral branches.

The LMCA passes between the main pulmonary artery and the left atrial appendage before bifurcating into the LAD and LCX arteries. The LAD descends within the anterior interventricular groove towards the cardiac apex, giving rise to septal perforator and diagonal branches that supply the anterior wall of the left ventricle, the apex and the anterior two-thirds of the interventricular septum. The LCX runs through the left atrioventricular groove, giving rise to obtuse marginal branches that primarily supply the lateral wall of the left ventricle.

Coronary dominance is defined by the vessel that gives rise to the PDA. It is classified as right dominant in 70–85% of individuals, left dominant in 5–15%, and codominant in 10–20% [3,4,5,6,7,8].

## 3. Epidemiology and Classification of CAA

The reported prevalence of coronary artery anomalies (CAAs) varies considerably across studies, primarily due to differences in their definition, classification and detection methods. Further variability arises from the largely asymptomatic nature of many CAAs, which often go undiagnosed unless cardiac imaging is specifically performed. Consequently, prevalence estimates differ not only between autopsy and imaging studies, but also between different imaging modalities.

In one of the largest angiographic series involving 126,595 patients undergoing invasive coronary angiography (ICA), CAAs were identified in 1.3% of cases. The most frequently reported anomalies were the left anterior descending (LAD) and left circumflex (LCX) arteries originating separately from the left sinus of Valsalva (30.4%), and the LCX originating from the right sinus of Valsalva or the right coronary artery (27.7%). It is important to note that myocardial bridging was not included in the definition of CAA due to its relatively high prevalence and frequent classification as a normal anatomical variant rather than a true coronary anomaly [9,10].

More recent studies using coronary computed tomography angiography (CCTA) have reported substantially higher prevalence rates than those based on conventional angiography. In a comparative study by Ghadri et al. involving 1759 CCTA patients and 9782 ICA patients, CAAs were identified in 7.9% and 2.1% of patients, respectively. Notably, the higher detection rate observed with CCTA persisted even after the exclusion of myocardial bridging, suggesting that the true prevalence of CAAs may have been underestimated in earlier angiographic studies. Furthermore, approximately 9% of the anomalies detected by CCTA were considered potentially serious, including those characterised by an interarterial course between the aorta and pulmonary artery. These findings highlight the superior diagnostic performance of CCTA for identifying and characterising CAAs [11].

In addition to its higher diagnostic yield, CCTA provides a detailed, three-dimensional visualisation of the origin of the coronary artery, its proximal course and its relationship with adjacent cardiovascular structures. This makes CCTA the imaging modality of choice for the anatomical assessment and risk stratification of coronary artery aneurysms (CAAs). Interestingly, prevalence estimates derived from autopsy studies generally range between 2% and 7%, which is more consistent with CCTA than ICA estimates.

Several classification systems have been proposed to categorise CAAs. Among these, the anatomical classification described by Gentile et al. remains one of the most widely adopted. This system categorises CAAs according to the affected coronary segment and divides them into anomalies of origin, course and termination (Table 2). Anomalies of origin are further subdivided into anomalous pulmonary origin, anomalous aortic origin, and congenital atresia of the left main coronary artery [12].

An alternative classification system, proposed by Rigatelli et al., takes a clinically oriented approach, categorising CAAs according to their potential to cause myocardial ischaemia, sudden cardiac death, or other adverse cardiovascular events. In this system, anomalies are classified as benign, relevant, severe or critical [13] (Table 3). While the anatomical classification remains useful for describing coronary morphology, the clinical classification provides a more practical framework for risk stratification and therapeutic decision-making. Consequently, the latter will be used throughout this review to discuss the clinical significance, pathophysiological implications and management of the various CAA subtypes.

## 4. Pathophysiology: Localization and Severity of Risk

### 4.1. Origin Anomalies

An origin anomaly is defined as an abnormally located coronary ostium. This may involve an anomalous origin from the aorta or pulmonary artery, or congenital atresia of the left main coronary artery (LMCA).

#### 4.1.1. Congenital Abnormalities of the Left Main Coronary Artery

Congenital abnormalities involving the left main coronary artery (LMCA) can be categorised into two distinct types.

The first is the relatively common anatomical variant of the left anterior descending (LAD) and left circumflex (LCX) arteries having separate origins from the left sinus of Valsalva (with a prevalence of 0.41–0.67%), which is generally considered to be benign with no clinical consequences.

**Figure 1:**
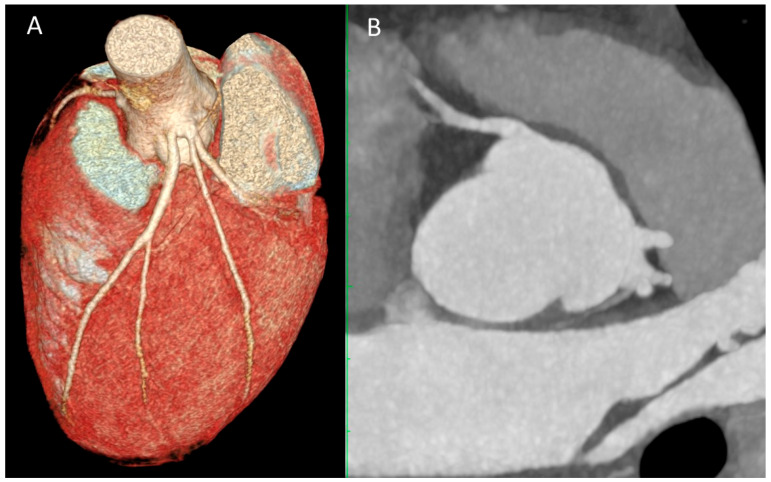
(**A**) Three-dimensional representation of an absent left main trunk or congenital atresia of the left main trunk. (**B**) CCTA image showing an absent left main trunk.

The second entity, which is much rarer, is congenital LMCA atresia or severe hypoplasia, which is characterised by the absence of a functional left main trunk. In these patients, collateral vessels may develop between the right and left coronary circulations. However, collateral flow is often insufficient to adequately perfuse the left ventricle. Consequently, affected individuals frequently develop myocardial ischaemia, left ventricular dysfunction, heart failure or sudden cardiac death during infancy or early childhood. Although uncommon, delayed diagnosis into adulthood has occasionally been reported in patients with extensive collateral circulation. Due to its potential to cause clinically significant myocardial ischaemia and adverse cardiovascular events, congenital LMCA atresia is classified as a Rigatelli Class II (‘relevant’) coronary anomaly, reflecting the need for careful clinical evaluation and management [12,14] (Figure 1).

#### 4.1.2. Anomalous Pulmonary Origin of the Coronary Arteries (APOCA)

**Figure 2:**
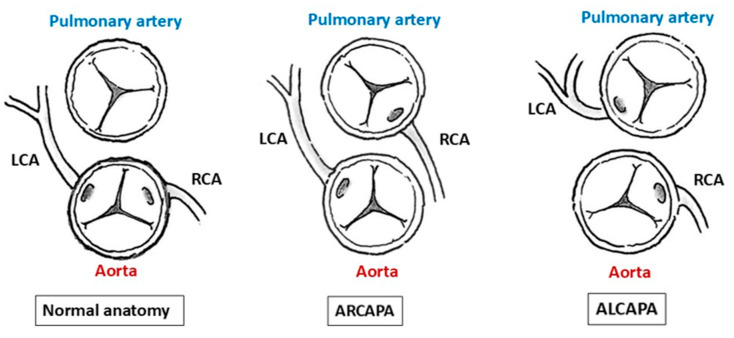
Anomalous pulmonary origin of the coronary arteries (APOCA). Schematic image illustrating the left coronary artery arising directly from the pulmonary artery (ALCAPA, also known as Bland-White-Garland syndrome) and the right coronary artery arising from the pulmonary artery (ARCAPA). RCA: Right coronary artery, LCA: Left coronary artery.

Anomalous pulmonary origin of the coronary arteries (APOCA) is a rare group of coronary anomalies, the clinical presentation and prognosis of which largely depend on the affected vessel and the extent of collateral circulation. The two main types are anomalous origin of the left coronary artery from the pulmonary artery (ALCAPA) and anomalous origin of the right coronary artery from the pulmonary artery (ARCAPA) (Figure 2).

ALCAPA, also known as Bland-White-Garland syndrome, occurs in around one in 300,000 live births and has a mortality rate of almost 90% in the first year of life if left untreated. During the neonatal period, elevated pulmonary vascular resistance maintains antegrade perfusion of the left coronary artery, meaning that affected infants are initially asymptomatic. However, as pulmonary arterial pressure decreases after birth, blood preferentially flows from the left coronary system into the pulmonary artery. This produces a coronary steal phenomenon and results in progressive myocardial ischaemia, left ventricular dysfunction and mitral regurgitation. Due to its severe clinical consequences and high mortality rate in the absence of surgical correction, ALCAPA is classified as a Rigatelli Class IV (“critical”) coronary anomaly [12,14,15,16].

In contrast, ARCAPA is less common and generally follows a more favourable clinical course. Most patients remain asymptomatic or present later in life with exertional angina, dyspnea, symptoms of heart failure, or, rarely, sudden cardiac death. As with ARCAPA, progressive collateralisation develops between the left and right coronary systems. This results in retrograde flow into the pulmonary artery and a coronary steal phenomenon. However, as myocardial perfusion of the left ventricle is preserved through the normally originating left coronary artery, ischaemic complications are less frequent than in ALCAPA. Consequently, ARCAPA is generally considered a Rigatelli Class III (‘severe’) coronary anomaly [3,17,18].

#### 4.1.3. Anomalous Aortic Origin of the Coronary Arteries (AAOCA)

Anomalous aortic origin of the coronary arteries (AAOCA) is a heterogeneous group of anomalies that includes a single coronary artery, inverted coronary arteries, anomalous origin of the left anterior descending (LAD) or left circumflex (LCX) artery from the right coronary artery, and anomalous coronary artery arising from the opposite sinus (ACAOS). Of these, ACAOS is the most clinically relevant subgroup due to its association with myocardial ischaemia and sudden cardiac death.

ACAOS includes the anomalous origin of the left coronary artery from the right sinus of Valsalva (AAOLCA), the anomalous origin of the right coronary artery from the left sinus of Valsalva (AAORCA), and the anomalous origin of the left circumflex artery (LCX) from the right sinus of Valsalva. The latter is generally considered to be a benign variant, unless it is associated with concomitant atherosclerotic disease. Consequently, clinical attention has primarily focused on AAOLCA and AAORCA [3,5,19].

**Figure 3:**
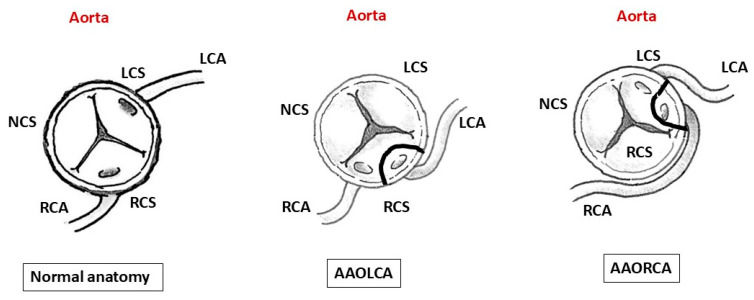
Anomalous aortic origin of the coronary arteries (AAOCA). Schematic image illustrating the left coronary artery arising directly from the right coronary sinus (AAOLCA) and the right coronary artery arising directly from the left coronary sinus (AAORCA). RCA: right coronary artery, LCA: Left coronary artery, anomalous origin of LCA from RCS (AAOLCA), anomalous origin of the RCA from LCS (AAORCA). LCS: Left coronary sinus, RCS: Right coronary sinus, NCS: Non-coronary sinus.

**Figure 4:**
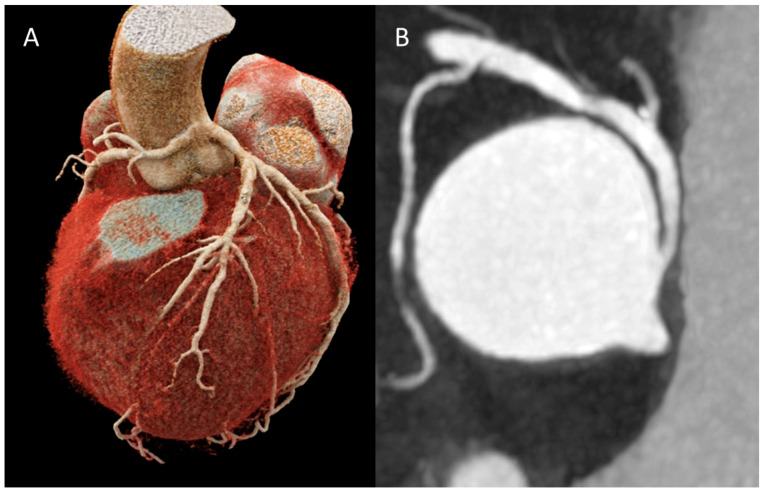
Anomalies of aortic origin: (**A**) Three-dimensional projection of the right coronary artery arising from the left sinus of Valsalva (**B**) CCTA image of an anomalous aortic origin of the right coronary artery from the left sinus of Valsalva (AAORCA).

AAORCA is more prevalent (0.03–0.92%) than AAOLCA (approximately 0.03%), although the latter is associated with a substantially higher risk of adverse events (Figure 3 and Figure 4). Both anomalies have been implicated in exercise-related sudden cardiac death. In a landmark study of 1866 sudden deaths among competitive athletes in the United States, coronary anomalies accounted for around 17% of cases, with AAOLCA being the anomaly most frequently identified. Accordingly, both entities are classified as Rigatelli Class III lesions.

**Figure 5:**
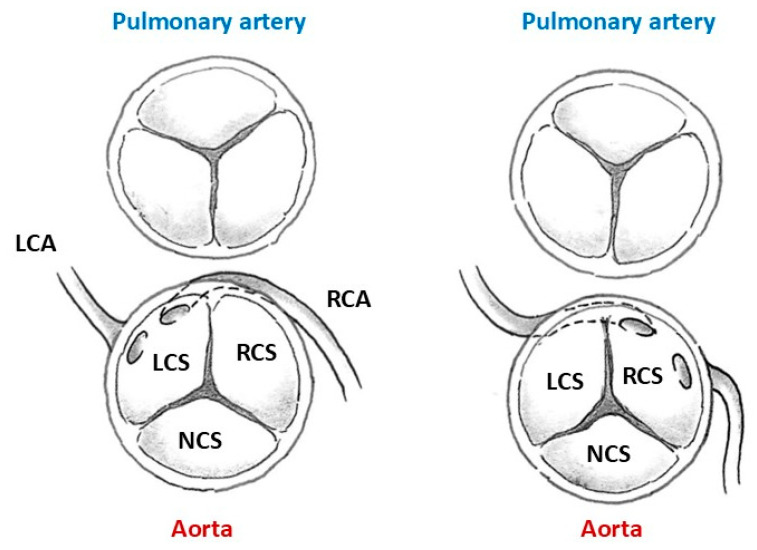
The interarterial course, especially with an intramural run (the coronary artery runs through the aortic wall, sharing the tunica media with the aorta), makes the artery prone to compression, particularly during systole.

The clinical consequences of ACAOS are largely determined by the course of the anomalous vessel. Five major anatomical pathways have been identified (Figure 4): interarterial, prepulmonic, subpulmonic (transseptal), retroaortic and retrocardiac. Of these, the interarterial pathway carries the greatest risk of myocardial ischaemia and sudden cardiac death. This risk is attributed to several anatomical features, including an acute-angle takeoff, slit-like ostium, proximal narrowing, and intramural aortic course. In this configuration, the anomalous vessel shares the tunica media of the aortic wall and can become dynamically compressed, especially during exercise, when aortic expansion further reduces the diameter of the lumen (Figure 5). While the precise mechanisms are not fully understood, these anatomical features ultimately result in impaired coronary reserve and may manifest clinically as exertional angina, dyspnoea, syncope, arrhythmias, myocardial infarction or sudden cardiac death [1,3,5,17,20,21].

### 4.2. Course Anomalies

Course anomalies represent one of the most commonly encountered categories of coronary artery anomalies. This is largely attributable to the inclusion of myocardial bridges and coronary artery aneurysms, conditions that are substantially more prevalent than most other coronary abnormalities. Although some authors regard these entities as anatomical variants rather than true anomalies because of their relatively high prevalence in the general population, they continue to be included in most contemporary classification systems and will therefore be discussed in the present review.

#### 4.2.1. Myocardial Bridges

Myocardial bridges (MBs) are anatomical variants in which a segment of a coronary artery—most commonly the mid-left anterior descending artery (LAD)—follows an intramyocardial course rather than its usual epicardial trajectory. The overlying muscle fibres constitute the myocardial bridge, while the intramyocardial arterial segment is referred to as the tunnelled artery.

For many years, MBs were considered benign because coronary perfusion predominantly occurs during diastole, while arterial compression is limited to systole. Consequently, systolic narrowing was initially thought to have minimal impact on myocardial blood flow. However, subsequent studies have demonstrated that the clinical significance of MBs varies considerably depending on multiple anatomical and haemodynamic factors, including the depth, length, and degree of compression of the tunnelled segment. To facilitate risk stratification, Angelini et al. proposed a severity score incorporating the extent of systolic narrowing and the length of the bridged segment (Table 4). This score was subsequently integrated into the Rigatelli classification, which distinguishes between lower-risk (Class I) and higher-risk (Class III) myocardial bridges. Although the relationship between anatomical characteristics and clinical outcomes remains incompletely defined, these parameters continue to influence diagnostic evaluation and therapeutic decision-making [22,23,24].

The pathophysiological consequences of MBs arise from the dynamic compression of the tunnelled artery during systole, coupled with delayed vessel relaxation in the early stages of diastole. Angiographic and intravascular ultrasound (IVUS) studies have demonstrated persistent narrowing that extends into diastole. This reduces coronary reserve and impairs myocardial perfusion, particularly within the subendocardium. These changes may manifest clinically as angina, exertional dyspnea, myocardial ischaemia, arrhythmias, stress cardiomyopathy or, rarely, sudden cardiac death. The characteristic angiographic appearance of narrowing during systole followed by expansion during diastole is known as the ‘milking phenomenon’ [23,25].

#### 4.2.2. Coronary Artery Aneurysms and Ectasia

**Figure 6:**
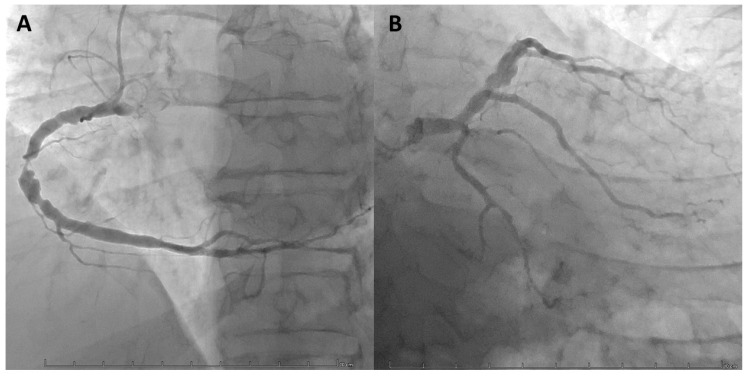
Coronarography images demonstrating focal coronary artery aneurysms localised on RCA (**A**) and LAD (**B**). Coronary aneurysms are defined as a focal dilatation of a coronary exceeding 1.5 times the diameter of the adjacent normal segment but involving less than 50% of the vessel length.

Coronary artery aneurysms (CAAs) are defined as focal dilatations that exceed 1.5 times the diameter of the adjacent normal vessel, but involve less than 50% of its length. By contrast, coronary artery ectasia refers to diffuse dilatation affecting more than 50% of the coronary artery (Figure 6).

Coronary aneurysms can be categorised as either saccular or fusiform in morphology, with the latter being the more prevalent form. They are reported to affect approximately 1–5% of patients undergoing coronary angiography. While the pathophysiology underlying ischaemic symptoms is not fully understood, altered flow dynamics, turbulent blood flow and thrombus formation within the aneurysmal segment are believed to contribute to myocardial ischaemia and distal embolisation. In adults, atherosclerosis is the most common cause, whereas Kawasaki disease is the main cause in children. Depending on their size, location and associated complications, coronary aneurysms can range from incidental findings to clinically significant lesions that are associated with myocardial ischaemia, acute coronary syndromes or, rarely, rupture. According to the Rigatelli classification, most aneurysms and ectatic lesions are categorised as clinically relevant (Class II) anomalies, although larger or symptomatic lesions may carry greater clinical significance [12,21,26,27].

### 4.3. Termination Anomalies

Termination anomalies encompass a spectrum of conditions characterised by abnormal communications or interruptions in the coronary circulation. The most significant of these are coronary artery fistulas (CAFs). CAFs are defined as abnormal connections between a coronary artery and a cardiac chamber, the coronary sinus, a major blood vessel, or another vascular structure. They are reported to affect between 0.1% and 0.2% of the general population, and most individuals remain asymptomatic throughout life. Around 90% of fistulas drain into the right-sided cardiac chambers or vessels, though left-sided drainage has also been documented. Due to their potential to cause clinically significant haemodynamic consequences despite often remaining asymptomatic, CAFs are generally classified as Rigatelli Class II (‘relevant’) coronary anomalies [5,28].

The main pathophysiological consequence of CAFs is the coronary steal phenomenon, whereby blood is diverted from the myocardial circulation towards a receiving chamber or vessel with a lower resistance. This can result in chronic volume overload and altered flow dynamics, leading to progressive dilatation and tortuosity of the feeding coronary artery. This can occasionally result in the development of giant fistulas, which are generally defined as those exceeding 8 mm in diameter. Single or multiple fistulas may coexist in the same patient. Potential complications include thrombosis, aneurysmal dilatation, stenosis, myocardial ischaemia, ventricular dysfunction, heart failure, infective endocarditis, rupture and, in rare cases, cardiogenic shock.

**Figure 7:**
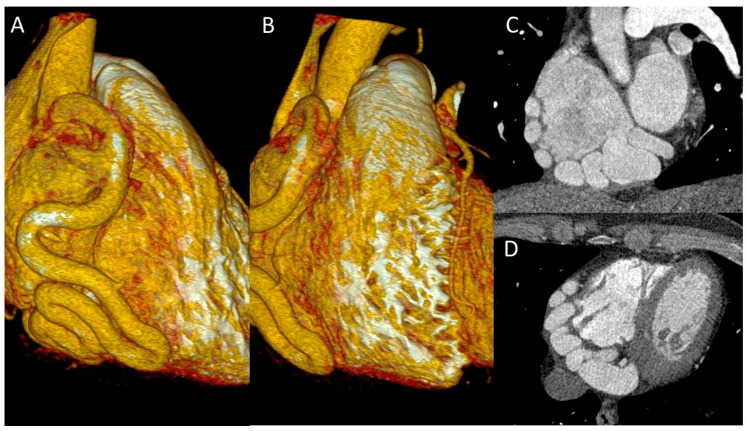
Coronary arteriovenous fistula: Circumflex Artery draining directly into the Coronary Sinus. (**A**,**B**) Three-dimensional volume-rendered image showing the circumflex artery giving rise to an arteriovenous fistula draining directly into the coronary sinus. (**C**,**D**) CCTA image demonstrating the course of the circumflex artery–to–coronary sinus fistulous connection.

The clinical presentation varies considerably according to patient age and fistula size. Signs of heart failure secondary to significant left-to-right shunting are typically seen in neonates and infants, whereas adults more commonly develop exertional angina, myocardial ischaemia, arrhythmias, pulmonary hypertension, heart failure, infective endocarditis or, rarely, sudden cardiac death. Symptomatic presentation most frequently occurs during the fifth and sixth decades of life [5,28] (Figure 7).

## 5. Management and Treatment

Accurate anatomical and functional assessment is fundamental to the management of coronary artery anomalies (CAAs). Beyond establishing the diagnosis, cardiac imaging plays a central role in risk stratification, identification of high-risk anatomical features, and therapeutic decision-making. Imaging findings help determine the likelihood of myocardial ischemia, arrhythmias, and sudden cardiac death (SCD), thereby guiding the need for conservative management, further functional testing, or intervention.

Coronary computed tomography angiography (CCTA) is currently considered the imaging modality of choice for the evaluation of CAAs. Its excellent spatial resolution enables precise characterisation of the origin, course, and termination of anomalous coronary arteries, as well as their relationships with adjacent cardiovascular structures. Consequently, CCTA has become the cornerstone of both diagnosis and anatomical risk assessment.

Additional imaging modalities provide complementary structural and functional information. Cardiac magnetic resonance (CMR) imaging allows assessment of ventricular function, myocardial viability, fibrosis, and ischemic burden, while invasive coronary angiography (ICA) remains useful in selected patients requiring hemodynamic assessment or concomitant evaluation of coronary artery disease.

Functional assessment of myocardial ischemia may be performed using exercise stress testing, stress echocardiography, stress CMR, or nuclear myocardial perfusion imaging (SPECT/PET), particularly in patients with anomalous aortic origin of a coronary artery (AAOCA), myocardial bridging, or symptoms suggestive of ischemia. These modalities help determine the physiological significance of anatomical findings and contribute to treatment selection.

Echocardiography remains particularly valuable in paediatric populations because of its non-invasive nature, widespread availability, and ability to identify major coronary abnormalities during routine evaluation. In addition, it plays an important role in the diagnosis and follow-up of anomalies such as ALCAPA, coronary artery fistulas, and coronary aneurysms.

Additionally, intravascular ultrasound (IVUS) has established itself as a valuable adjunct in the management of CAA, particularly in cases of anomalies with an interarterial course. According to both Lopez and Angelini, IVUS is most useful when a definitive quantification of stenosis severity is clinically indicated, and when planning or executing stent angioplasty by providing precise vessel sizing, lesion characterisation, and real-time guidance that conventional angiography cannot offer. However, its use should be reserved for expert centres, as it requires significant operator expertise, and it intervenes only after non-invasive imaging modalities such as cardiac MRI or CTA have established the primary diagnosis [29,30].

Although emerging techniques such as FFR-CT may hold promise for the evaluation and characterisation of coronary artery anomalies and their hemodynamic impact, the current literature remains scarce, consisting largely of isolated case reports. Further studies are therefore needed before such modalities can be reliably integrated into standard clinical practice.

Given the heterogeneity of CAAs and their associated risks, contemporary management strategies rely on the integration of anatomical findings, functional assessment, patient symptoms, and individual risk factors. Table 5 and Table 6 summarise current guideline recommendations for the diagnostic evaluation and management of CAAs [3,7,12,31].

**Figure 8:**
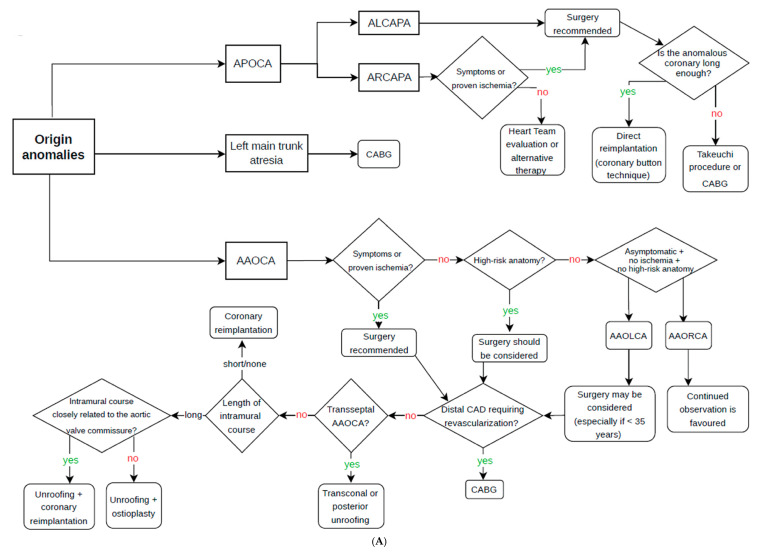

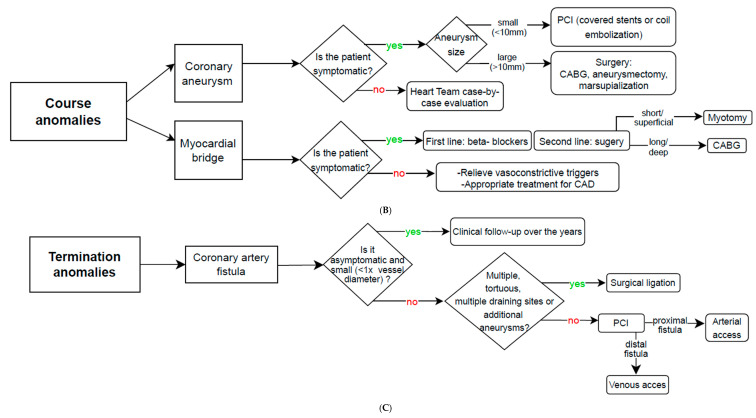
Expert-synthesised decision tree, based on current literature, for selection of recommended and/or suggested treatment modalities in patients with coronary artery anomalies. (**A**) Origin anomalies. (**B**) Course anomalies. (**C**) Termination anomalies. AAOCA: Anomalous aortic origin of the coronary arteries, ALCAPA: Anomalous left coronary artery from the pulmonary artery, APOCA: Anomalous pulmonary origin of the coronary arteries, ARCAPA: Anomalous right coronary artery from the pulmonary artery, CABG: Coronary artery bypass graft, PCI: Percutaneous coronary intervention.

Based on the available literature, contemporary guideline recommendations and the evidence reviewed herein, a simplified management algorithm for CAAs is proposed in Figure 8. This algorithm takes into account anomaly subtype, symptom status, objective evidence of ischaemia, high-risk anatomical features and lesion morphology in order to guide treatment selection, ranging from conservative surveillance to percutaneous or surgical intervention.

### 5.1. Origin Anomalies (Figure 8A)

#### 5.1.1. Anomalous Aortic Origin of the Coronary Arteries (AAOCA)Indications for Intervention

##### Indications for Intervention

Both European and American guidelines recommend surgical intervention for symptomatic patients with anomalous aortic origin of the coronary arteries (AAOCA), or for those with objective evidence of myocardial ischaemia. However, there are differences in recommendations for the management of asymptomatic individuals and the role of high-risk anatomical features.

For the anomalous origin of the left coronary artery from the right sinus of Valsalva (AAOLCA), both societies generally support surgical correction, regardless of symptoms, due to the well-established link with sudden cardiac death (SCD). In contrast, recommendations for the anomalous origin of the right coronary artery from the left sinus of Valsalva (AAORCA) are more conservative, particularly in the absence of symptoms or inducible ischaemia. The American guidelines also specifically include ventricular arrhythmias as an indication for surgery.

High-risk anatomical features recognised by both societies include an inter-arterial or intramural course, a slit-like ostium, proximal narrowing and an acute-angle take-off. Nevertheless, the absolute risk of SCD remains difficult to quantify, and the benefit of prophylactic surgery in asymptomatic patients without demonstrable ischaemia remains uncertain. Consequently, management should incorporate clinical presentation, anatomical characteristics, age, level of physical activity, and patient preferences [4,18,19,34,35,36,37,38].

##### Surgical Techniques

The primary objective of surgical treatment is to eliminate the anatomical cause of dynamic coronary compression and impaired coronary reserve. The choice of procedure largely depends on the presence and extent of the intramural segment.

For patients with a long intramural course, unroofing is the most commonly performed procedure. This technique involves opening the shared wall between the aorta and the intramural coronary artery to create a new coronary orifice within the appropriate sinus of Valsalva. If a slit-like or stenotic ostium is present, ostioplasty can be performed simultaneously using a pericardial patch to enlarge the coronary origin.

**Figure 9:**
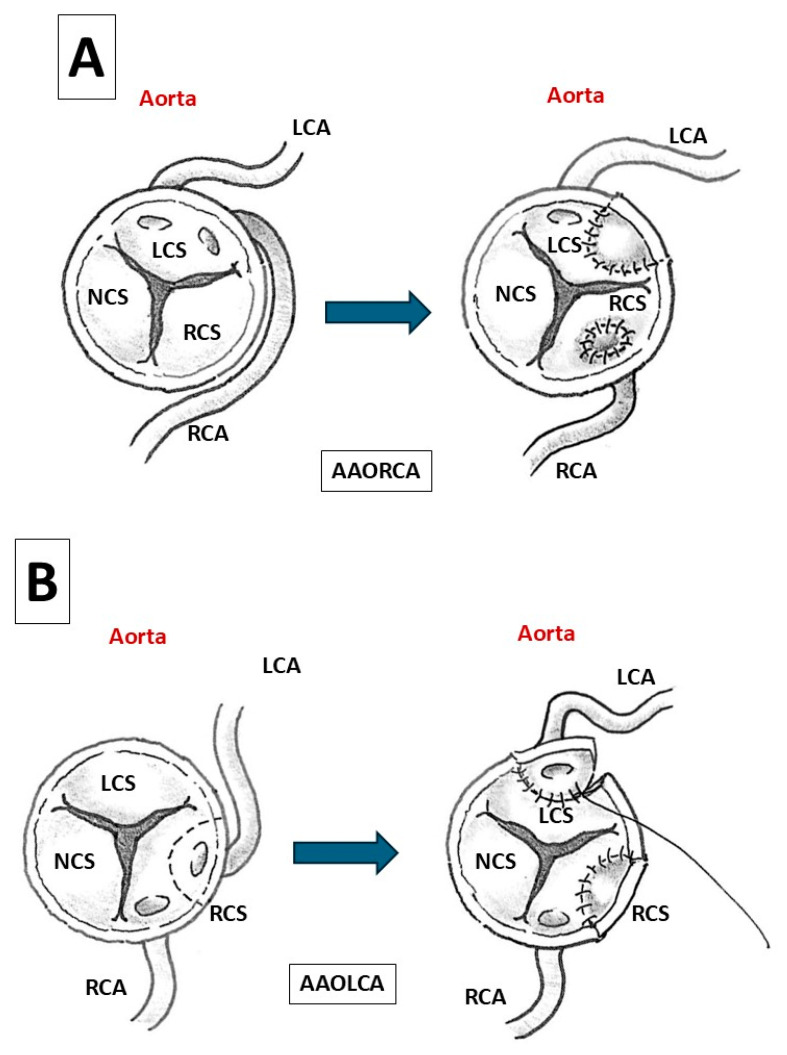
Ostial translocation as a surgical technique for anomalous coronary artery origins. Ostial translocation is a surgical method used to correct anomalous origins of the coronary arteries related to abnormal aortic sinus origin. (**A**) Ostial translocation in AAORCA: relocation of the right coronary artery (RCA) from the left coronary sinus (LCS) to the right coronary sinus (RCS). (**B**) Ostial translocation in AAOLCA: relocation of the left coronary artery (LCA) from the right coronary sinus (RCS) to the left coronary sinus (LCS).

Coronary reimplantation is generally preferred when the intramural segment is short or absent. In this procedure, the anomalous coronary artery is detached from its abnormal origin and reimplanted into the appropriate sinus. Although this approach is technically more demanding, it restores normal coronary anatomy and avoids manipulation of the aortic commissures (Figure 9A,B).

Historically, unroofing has been considered the standard surgical treatment for intramural AAOCA due to its technical simplicity and favourable early outcomes. However, emerging evidence suggests that coronary reimplantation may provide superior long-term results. A recent single-centre study of 230 patients found that reoperation was significantly more frequent after unroofing (5.8%) than after coronary reimplantation (0%), primarily due to recurrent ischaemia, residual stenosis or complications related to commissural manipulation. Consequently, some experienced centres have increasingly adopted coronary reimplantation as their preferred primary strategy for suitable patients [4,18,19,34,35,36,37,38].

In selected patients with an interarterial course, pulmonary artery translocation may be considered to increase the distance between the great vessels and reduce external coronary compression. This procedure can be performed on its own or alongside unroofing.

The management of AAOCA with a transseptal course depends on the depth of the intramyocardial segment. Superficial transseptal segments can be treated via a posterior approach after temporary pulmonary artery transection. Deeper segments can be addressed using transconal (infundibular) unroofing through the right ventricular outflow tract, followed by reconstruction with a pericardial patch [31,34,36,39,40].

##### Coronary Artery Bypass Grafting

Coronary artery bypass grafting (CABG) is typically recommended for older patients or individuals with concomitant atherosclerotic coronary artery disease. It is also used in cases where anatomical repair is not technically feasible. While CABG avoids direct manipulation of the anomalous coronary artery, long-term graft patency may be compromised by competitive flow through the native vessel [12,31,36,38].

##### Percutaneous Coronary Intervention

The role of percutaneous coronary intervention (PCI) is still unclear. Current evidence is restricted to case reports and small observational studies. While PCI can alleviate symptoms in certain adult patients, it does not address the underlying anatomical abnormality. There are also ongoing concerns about restenosis and stent durability in vessels subject to dynamic mechanical compression. Currently, PCI should only be considered in carefully selected patients who are poor surgical candidates or who have concomitant atherosclerotic disease requiring intervention [3,31,41,42].

#### 5.1.2. Anomalous Pulmonary Origin of the Coronary Arteries (APOCA)

American and European guidelines largely agree on the management of anomalous pulmonary origin of the coronary arteries (APOCA). Surgical correction is recommended for all patients with ALCAPA (anomalous left coronary artery from the pulmonary artery), regardless of symptoms, due to the high risk of myocardial ischaemia, left ventricular dysfunction and sudden cardiac death. For patients with an anomalous right coronary artery arising from the pulmonary artery (ARCAPA), surgery is recommended for symptomatic individuals and is also considered for asymptomatic patients with evidence of myocardial ischaemia or ventricular dysfunction [7,43,44,45,46].

**Figure 10:**
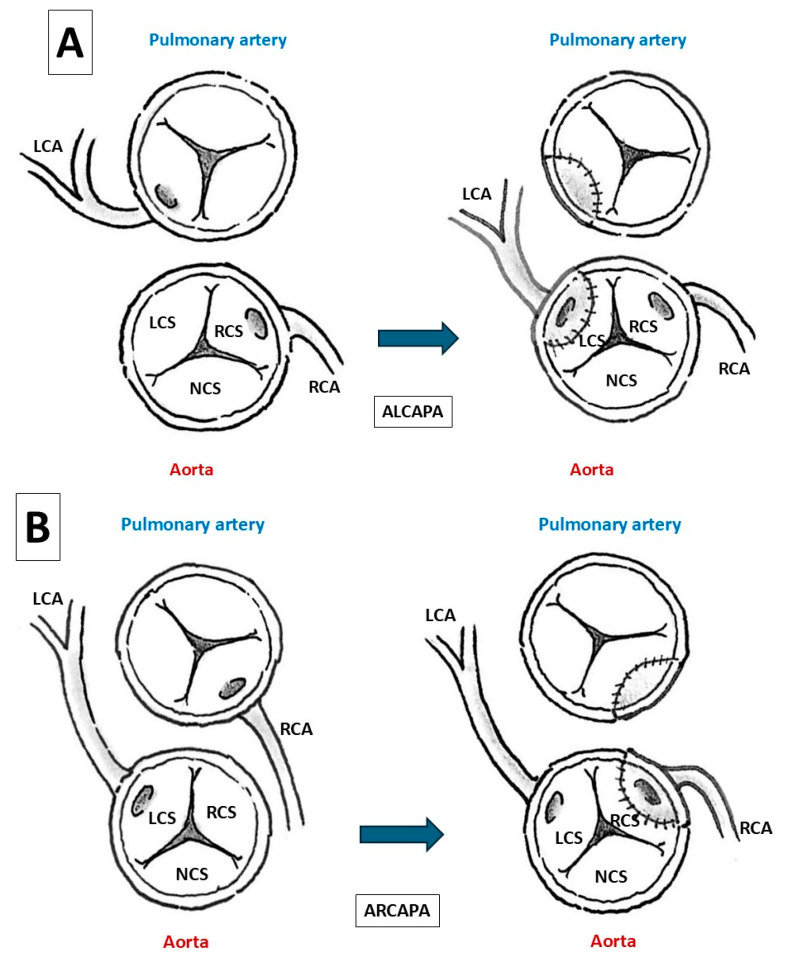
The preferred technique for pulmonary origins of coronary arteries is direct reimplantation of the coronary artery in the correct sinus (coronary button technique). A circular portion of the PA around the anomalous coronary ostium (hence the name button) is taken and implanted in the correct site of the aorta. Direct implantation of LCA in ALCAPA (**A**) and RCA in ARCAPA (**B**).

The primary objective of surgery is to restore a two-coronary arterial system that can supply oxygenated blood to the entire myocardium. Direct coronary reimplantation (coronary button technique) is currently considered the preferred surgical strategy whenever anatomically feasible (Figure 10A,B). This technique involves the excision of the anomalous coronary ostium together with a cuff of pulmonary arterial wall, followed by reimplantation into the appropriate aortic sinus. This restores normal coronary anatomy and physiology.

If direct reimplantation is not technically feasible due to insufficient coronary length or unfavourable anatomy, the Takeuchi procedure may be considered. This operation involves creating an intrapulmonary tunnel that connects the anomalous coronary ostium to a surgically created aortopulmonary window. This establishes a source of oxygenated blood flow to the affected coronary artery.

Coronary artery bypass grafting (CABG) with ligation of the anomalous coronary origin is an alternative option, particularly for adult patients or in cases where anatomical repair is not possible. Although contemporary surgical outcomes are excellent, the risk of surgery is still influenced by factors such as the patient’s age, the degree of left ventricular dysfunction and the severity of associated mitral regurgitation. Following the successful restoration of dual coronary circulation, most patients show significant improvement in left ventricular function and a reduced risk of adverse cardiac events, including sudden cardiac death [7,43,44,45,46].

#### 5.1.3. Atresia Left Main Trunk

As congenital left main coronary artery (LMCA) atresia often leads to significant myocardial ischaemia and progressive left ventricular dysfunction, surgical correction is usually recommended once the diagnosis has been confirmed. The aim of the treatment is to restore adequate blood supply to the left coronary territory and prevent irreversible damage to the ventricles.

The most commonly reported surgical strategy is coronary artery bypass grafting using either the internal mammary artery or a saphenous vein graft, or both when a single conduit is insufficient to achieve complete revascularisation. Although experience remains limited due to the rarity of this anomaly, published case series and reports have consistently demonstrated favourable surgical outcomes, with substantial symptomatic improvement and recovery of myocardial function following revascularisation [1,5,47,48].

### 5.2. Course Anomalies (Figure 8B)

#### 5.2.1. Myocardial Bridges

The management of myocardial bridges (MBs) remains challenging and is largely determined by symptom burden, objective evidence of ischaemia and anatomical characteristics, such as the depth, length and degree of systolic compression of the tunnelled segment.

Asymptomatic patients generally require no specific intervention and are managed conservatively through the modification of risk factors and the optimisation of cardiovascular prevention strategies. For symptomatic patients, medical therapy is the primary treatment. Beta-blockers are preferred as their negative chronotropic and inotropic effects prolong diastolic filling time and reduce the degree of systolic compression. Non-dihydropyridine calcium-channel blockers may be an alternative option, particularly for patients with coronary vasospasm. In contrast, nitrates are generally avoided as they can exacerbate systolic compression by increasing vessel wall compliance and reflex sympathetic activation [22,23,24,25,49].

In patients with persistent symptoms or documented ischaemia despite receiving optimal medical therapy, invasive treatment may be considered. However, the role of percutaneous coronary intervention (PCI) remains controversial due to concerns regarding in-stent restenosis, stent fracture and the limited availability of long-term outcome data. Surgical treatment is therefore preferred for carefully selected patients. Surgical myotomy, involving the division of overlying myocardial fibres, is generally favoured for superficial bridges. However, coronary artery bypass grafting (CABG) may be considered for patients with long, deep or anatomically complex bridged segments [22,23,24,25,49].

#### 5.2.2. Coronary Artery Aneurysms and Ectasia

The management of coronary artery aneurysms and ectasia should be tailored to the individual patient and guided by symptoms, aneurysm size and location, the underlying cause, the rate of expansion, and the presence of other coronary artery diseases. Although thrombus formation and distal embolisation are important concerns, the optimal antithrombotic strategy remains uncertain due to conflicting evidence and the absence of randomised controlled trials.

Invasive treatment is generally reserved for symptomatic patients or those with large aneurysms, progressive enlargement, complications or associated obstructive coronary artery disease. Percutaneous approaches are most commonly used for smaller aneurysms and include covered stent implantation or coil embolisation. Surgical treatment is generally preferred for larger aneurysms, giant aneurysms, rapidly enlarging lesions or patients requiring concomitant coronary revascularisation. Available surgical options include aneurysm ligation, aneurysmectomy, marsupialisation and coronary artery bypass grafting (CABG), with ligation combined with CABG being one of the most frequently employed strategies [26,40,50,51,52].

### 5.3. Termination Anomalies (Figure 8C)

The management of coronary artery fistulas (CAFs) is primarily determined by fistula size, symptom burden, haemodynamic significance and the presence of complications. Small fistulas, which are generally defined as having a diameter smaller than the largest unaffected coronary artery, may close spontaneously and are therefore usually managed conservatively, with regular clinical and imaging follow-ups in line with current European guidelines.

For medium and large fistulas (defined as having a diameter of 1–2 and >2 times the reference coronary artery diameter, respectively), intervention is recommended if symptoms are present, there is significant left-to-right shunting, ventricular dysfunction, myocardial ischaemia, or other complications. The 2018 American guidelines also recommend that these patients be evaluated by a multidisciplinary Heart Team to guide management [28,32,33,53,54].

The primary objective of treatment is to achieve complete closure of the fistulous communication and eliminate the associated coronary steal phenomenon. Percutaneous transcatheter closure is currently considered the preferred treatment strategy for most anatomically suitable fistulas. Device selection and access route depend on fistula morphology, with arterial access generally favoured for proximally originating fistulas and venous access more commonly used for distal lesions.

Surgical treatment is reserved for complex fistulas, including large, tortuous or multiple fistulas, those associated with aneurysmal dilatation, and those draining into multiple sites. In such cases, the most commonly employed technique is surgical ligation of the fistulous connection, which is often performed at the drainage site [28,32,33,53,54].

## 6. Conclusions

Coronary artery anomalies (CAAs) encompass a broad and heterogeneous spectrum of congenital coronary abnormalities whose clinical significance ranges from incidental anatomical variants to lesions associated with myocardial ischemia, ventricular dysfunction, and sudden cardiac death. Although traditionally considered rare, the increasing use of coronary computed tomography angiography has revealed that several anomalies are more prevalent than previously recognised, highlighting the importance of distinguishing benign anatomical variants from clinically significant disease.

One of the principal challenges in the field remains the marked heterogeneity of CAAs. While anatomical classifications facilitate morphological description, their ability to guide clinical management is limited. Conversely, clinically oriented classifications better reflect the variable prognostic implications of individual anomalies. Ultimately, neither anatomy nor symptoms alone is sufficient to determine risk. Rather, management must integrate anatomical characteristics, physiological consequences, objective evidence of ischemia, patient age, and clinical presentation.

Among all CAAs, anomalies of coronary origin, particularly anomalous aortic origin of a coronary artery (AAOCA) and anomalous origin from the pulmonary artery, have been the most extensively investigated and consequently represent the foundation of current guideline recommendations. The identification of high-risk anatomical features-including an interarterial or intramural course, slit-like ostium, and acute-angle takeoff-has significantly improved risk stratification and surgical decision-making. In contrast, course and termination anomalies remain comparatively understudied despite their association with myocardial ischemia, heart failure, arrhythmias, thromboembolic complications, and other adverse cardiovascular events.

Advances in multimodality imaging have fundamentally changed the diagnostic approach to CAAs. Contemporary management increasingly relies on the combination of anatomical assessment by coronary computed tomography angiography and functional evaluation of myocardial ischemia using stress imaging modalities. This integrated approach has allowed a transition from purely anatomy-based decision-making toward individualised risk assessment and patient-specific treatment strategies.

Despite substantial progress, important knowledge gaps persist. Current recommendations are largely derived from observational studies, registries, and expert consensus, while prospective data and long-term outcome studies remain limited. Future research should focus on refining risk stratification models, clarifying the natural history of less common anomalies, and establishing evidence-based thresholds for intervention. Such efforts will be essential to further standardise management and optimise outcomes across the entire spectrum of coronary artery anomalies.

## Figures and Tables

**Table 1:** Key features of coronary anatomy described by Angelini et al. [1] for identifying a coronary artery as normal.

Feature	Range
**No. of ostia**	2 to 4
**Location**	Right and left anterior sinuses (upper midsection)
**Proximal orientation**	45° to 90° off the aortic wall
**Proximal common stem or trunk**	Only left (LAD and Cx)
**Proximal course**	Direct, from ostium to destination
**Mid-course**	Extramural (subepicardial)
**Branches**	Adequate for the dependent myocardium
**Essential territories**	RCA (RV free wall), LAD (anteroseptal), OM (LV free wall)
**Termination**	Capillary bed

**Table 2:** Anatomical classification of coronary artery anomalies proposed by Gentile et al. There are mainly three categories, entitled as origin, course and termination anomalies [12].

**Origin** **anomalies**	Anomalous pulmonary origin of the coronary arteries (APOCA)	Anomalous origin of the left coronary artery from the pulmonary artery (ALCAPA)
		Anomalous origin of the right coronary artery from the pulmonary artery (ARCAPA)
		Anomalous origin of the circumflex artery from the pulmonary artery
		Total anomalous origin of the coronary arteries from the pulmonary artery (TCAPA)
	Anomalous aortic origin of the coronary arteries (AAOCA)	Anomalous origin of the left main coronary artery from the right sinus of Valsalva
		Anomalous origin of the right coronary artery from the left sinus of Valsalva (AAORCA)
		Anomalous origin of the left anterior descending coronary artery from the right sinus of Valsalva (AAOLCA)
		Anomalous origin of the circumflex artery from the right sinus of Valsalva
		Anomalous origin of the left anterior descending artery from the right coronary artery
		Anomalous origin of the circumflex artery from the right coronary artery
		Single coronary artery
		Inverted coronary arteries
		Others
	Congenital atresia of the left main trunk (absent left main trunk)	
**Course** **anomalies**	Myocardial (or coronary) bridge	Symptomatic-asymptomatic
	Coronary aneurysm	Congenital or acquired
**Termination anomalies**	Coronary arteriovenous fistula (CAF)	Congenital or acquired
	Coronary stenosis	Congenital or acquired

**Table 3:** Clinical significance classification proposed by Rigatelli et al. There are four main categories, going from benign to critical [13].

Class	Coronary Artery Anomaly
**I. Benign**	- Ectopic origin of LCx from right sinus - Separate origin of LCx and LAD - Ectopic origin of LCx from the RCA - Dual LAD types I-IV - Myocardial bridge (score ≤ 5)
**II. Relevant** **Related to myocardial ischemia**	- Coronary artery fistula - Single coronary artery R-L, I-II-III, A-P - Ectopic origin of LCA from PA - Atretic coronary artery - Hypoplastic coronary artery
**III. Severe** **Potentially related to sudden death**	- Ectopic origin of LCA from the right sinus - Ectopic origin of RCA from the left sinus - Ectopic origin of RCA from the PA - Single coronary artery R-L, I-II-III B - Myocardial bridge (score ≥ 5)
**IV. Critical** **Related to sudden death/myocardial ischemia and associated with superimposed CAD**	- Class II and superimposed CAD - Class III and superimposed CAD

**Table 4:** Myocardial bridges (MBs) score system: severity scoring for MBs, varying from 2 to 5, based on systolic narrowing and segment length, as described by Angelini and used in Rigatelli’s classification [22].

**Systolic Narrowing**	**Rating**
<50%	1
50–75%	2
>75%	3
**Stenotic Segments (Length)**	
<1 cm	1
>1 cm	2

**Table 5:** Comparison of international guidelines for the evaluation of CAAs [32,33] AHA/ACC: American Heart Association/American College of Cardiology, ESC: European Society of Cardiology, CT: computed tomography, CMR: cardiac magnetic resonance, LD: Limited Data, COR: Class of Recommendation, and LOE: Level of Evidence.

2018 AHA/ACC Guideline for the Management of Adults with Congenital Heart Disease			2020 ESC Guidelines for the Management of Adult Congenital Heart Disease		
Recommendations	COR	LOE	Recommendations	COR	LOE
Coronary angiography, using catheterization, CT, or CMR, is recommended for evaluation of an anomalous coronary artery.	I	C-LD	Nonpharmacologic functional imaging (e.g., nuclear study, echocardiography, or CMR with physical stress) is recommended in patients with coronary anomalies to confirm/exclude myocardial ischemia.	I	C
Anatomic and physiologic evaluation should be performed in patients with anomalous aortic origin of the left coronary from the right sinus and/or right coronary from the left sinus.	I	C-LD			

**Table 6:** Comparison of international guidelines for the surgical treatment of origin CAAs. The bold horizontal line separates recommendations concerning anomalies of aortic origin (above) from those concerning pulmonary origins (below) [32,33]. AAOC: anomalous aortic origin of the coronaries, AAOLCA: anomalous aortic origin of the left coronary artery, AAORCA: anomalous aortic origin of the right coronary artery, ALCAPA: anomalous left coronary from the pulmonary artery, ARCAPA: anomalous right coronary from the pulmonary artery, NR: Non-Randomised, EO: Expert Opinion, LD: Limited Data, COR: Class of Recommendation and LOE: Level of Evidence.

2018 AHA/ACC Guideline for the Management of Adults with Congenital Heart Disease			2020 ESC Guidelines for the Management of Adult Congenital Heart Disease		
Recommendations	COR	LOE	Recommendations	COR	LOE
Surgery is recommended for AAOCA from the left sinus or AAOCA from the right sinus for symptoms or diagnostic evidence consistent with coronary ischemia attributable to the anomalous coronary artery.	I	B-NR	Surgery is recommended for AAOCA in patients with typical angina symptoms who present with evidence of stress-induced myocardial ischemia in a matching territory or high-risk anatomy.	I	C
Surgery is reasonable for an anomalous aortic origin of the left coronary artery from the right sinus in the absence of symptoms or ischemia.	IIa	C-LD	Surgery should be considered in asymptomatic patients with AAOCA (right or left) and evidence of myocardial ischemia.	IIa	C
Surgery for AAOCA is reasonable in the setting of ventricular arrhythmias.	IIa	C-EO	Surgery should be considered in asymptomatic patients with AAOLCA and no evidence of myocardial ischemia but a high-risk anatomy.	IIa	C
Surgery or continued observation may be reasonable for asymptomatic patients with an anomalous left coronary artery arising from the right sinus or right coronary artery arising from the left sinus without ischemia or anatomic or physiological evaluation suggesting potential for compromise of coronary perfusion (e.g., intramural course, fish-mouth-shaped orifice, acute angle).	IIb	B-NR	Surgery may be considered for symptomatic patients with AAOCA even if there is no evidence of myocardial ischaemia or high-risk anatomy.	IIb	C
			Surgery may be considered for asymptomatic patients with AAOLCA without myocardial ischemia and without high-risk anatomy when they present at a young age (<35 years).	IIb	C
			Surgery is not recommended for AAORCA in asymptomatic patients without myocardial ischemia and without high-risk anatomy.	III	C
Surgery is recommended for ALCAPA.	I	B-NR	Surgery is recommended for ALCAPA.	I	C
In a symptomatic adult with an anomalous right coronary artery from the PA with symptoms attributed to the anomalous coronary, surgery is recommended.	I	C-EO	Surgery is recommended in patients with ARCAPA and symptoms attributable to an anomalous coronary artery.	I	C
Surgery for the anomalous right coronary artery from the PA is reasonable in an asymptomatic adult with ventricular dysfunction or with myocardial ischemia attributed to an anomalous right coronary artery from the PA.	IIa	C-EO	Surgery should be considered for ARCAPA in asymptomatic patients with ventricular dysfunction or myocardial ischemia attributable to a coronary anomaly.	IIa	C

## Data Availability

The original contributions presented in this study are included in the article. Further inquiries can be directed to the corresponding authors.
